# Partition of energy for a dissipative quantum oscillator

**DOI:** 10.1038/s41598-018-34385-9

**Published:** 2018-10-31

**Authors:** P. Bialas, J. Spiechowicz, J. Łuczka

**Affiliations:** 1 Institute of Physics and Silesian Center for Education and Interdisciplinary Research, University of Silesia, 41-500 Chorzów, Poland; 2 Institute of Physics, University of Augsburg, D-86135 Augsburg, Germany

## Abstract

We reveal a new face of the old clichéd system: a dissipative quantum harmonic oscillator. We formulate and study a quantum counterpart of the energy equipartition theorem satisfied for classical systems. Both mean kinetic energy *E*_*k*_ and mean potential energy *E*_*p*_ of the oscillator are expressed as *E*_*k*_ = 〈*ε*_*k*_〉 and *E*_*p*_ = 〈*ε*_*p*_〉, where 〈*ε*_*k*_〉 and 〈*ε*_*p*_〉 are mean kinetic and potential energies per one degree of freedom of the thermostat which consists of harmonic oscillators too. The symbol 〈...〉 denotes two-fold averaging: (i) over the Gibbs canonical state for the thermostat and (ii) over thermostat oscillators frequencies *ω* which contribute to *E*_*k*_ and *E*_*p*_ according to the probability distribution ${{\mathbb{P}}}_{k}(\omega )$ and ${{\mathbb{P}}}_{p}(\omega )$, respectively. The role of the system-thermostat coupling strength and the memory time is analysed for the exponentially decaying memory function (Drude dissipation mechanism) and the algebraically decaying damping kernel.

## Introduction

In classical physics a harmonic oscillator describes small oscillations. Its quantum version is a standard model to introduce creation and annihilation Bose operators. In the theory of open quantum systems the harmonic oscillator is one of the simplest systems to investigate dissipation processes (see e.g.^1^ and refs therein) and decoherence phenomena (see e.g.^2,3^ and refs therein). It has attracted considerable interest over the last fifty years. Nevertheless there is still a plenty of room which is *terra incognita*. As an example, it has been lately applied in the problem of quantum-to-classical transition, formation of dynamical spectrum broadcast structures and classical objectivity as a property of quantum states^4^. Finally, we subjectively cite only a few papers^5–9^ published in the last two years to confirm that it is still the topic of active research. We also wish to revisit the dissipative quantum oscillator and discuss a quite different aspect, namely, the quantum counterpart of the theorem of energy equipartition (TEE) in classical statistical physics. Surely, it belongs to one of the fundamental laws which represents a universal relation in the sense that it does not depend on a number of particles in the system, a potential force which acts on them, any interaction between particles or the strength of coupling between the system and thermostat^10,11^. Beginning of its formulations is dated back to 19th century, to the times of James Clerk Maxwell and Ludwig Boltzmann. The latter in 1876 showed that average kinetic energy is equally shared in a portion of *E*_*k*_ = *k*_*B*_*T*/2 among all degrees of freedom of a system^12^. Since that time the TEE has become one of the most important and most useful relation exploited in various branches of Natural Science. In contrast, this law is no longer true for quantum systems. From the time of Max Planck and birth of quantum physics a quantum counterpart of TEE has not been explicitly proposed. Partial results on mean energy of some particular systems can be found in literature. Lately, we have derived an appealing formula which is a generalization of the classical TEE^13^. In this case the mean kinetic energy is not shared equally among all accessible degrees of freedom. In contrast, the kinetic energy *E*_*k*_ of a quantum harmonic oscillator is a thermally averaged kinetic energy per one degree of freedom of the thermostat oscillators. The latter contribute to *E*_*k*_ with different degrees: if the thermostat oscillator has eigenfrequency *ω* then its input to *E*_*k*_ is determined by the probability density ${{\mathbb{P}}}_{k}$(*ω*). We study the impact of two dissipation mechanism determined by the exponentially and algebraically decaying dissipation function on properties of the probability distribution ${{\mathbb{P}}}_{k}$(*ω*) and the mean kinetic energy of the quantum harmonic oscillator. Similar analysis is performed for the mean potential energy of the system.

## Model and Results

We study the celebrated model of a quantum open system *S*, i.e. a quantum harmonic oscillator of mass *M* and eigenfrequency *ω*_0_. It is in contact with a heat bath *B* modelled as a collection of independent quantum harmonic oscillators which form thermostat of temperature *T* being in an equilibrium Gibbs canonical state. The Hamiltonian of such a composite system *S* + *B* has the form^1,14–21^ (a complete list of papers concerning this problem is too long and our choice is selective)

1 $$H=\frac{{p}^{2}}{2M}+\frac{1}{2}M{\omega }_{0}^{2}{x}^{2}+\sum _{i}\,[\frac{{p}_{i}^{2}}{2{m}_{i}}+\frac{{m}_{i}{\omega }_{i}^{2}}{2}{({q}_{i}-\frac{{c}_{i}x}{{m}_{i}{\omega }_{i}^{2}})}^{2}],$$

where the coordinate and momentum operators {*x*, *p*} refer to the Brownian particle and {*q*_*i*_, *p*_*i*_} are the coordinate and momentum operators of the *i*-th heat bath oscillator of mass *m*_*i*_ and the eigenfrequency *ω*_*i*_. The parameter *c*_*i*_ characterizes the coupling strength of the central system *S* with the *i*-th thermostat oscillator. All coordinate and momentum operators obey canonical equal-time commutation relations. From the Heisenberg equations of motion for all coordinate and momentum operators one can obtain an effective equation of motion for the oscillator coordinate operator *x*(*t*). It is called a generalized quantum Langevin equation (GQLE) and reads^22^

2 $$M\ddot{x}(t)+M{\omega }_{0}^{2}x(t)+{\int }_{0}^{t}\,du\,\gamma (t-u)\dot{x}(u)=-\,\gamma (t)x\mathrm{(0)}+\eta (t),$$

where dot denotes time derivative, *γ*(*t*) is a dissipation function (damping or memory kernel) and *η*(*t*) is quantum noise,

3 $$\gamma (t)={\int }_{0}^{{\rm{\infty }}}d\omega \,J(\omega )\cos (\omega t),$$

4 $$\eta (t)=\sum _{i}\,{c}_{i}[{q}_{i}(0)\cos ({\omega }_{i}t)+\frac{{p}_{i}(0)}{{m}_{i}{\omega }_{i}}\,\sin ({\omega }_{i}t)]$$

and *J*(*ω*) is a spectral density of thermostat modes which contains all information on the system-thermostat coupling:

5 $$J(\omega )=\sum _{i}\,\frac{{c}_{i}^{2}}{{m}_{i}{\omega }_{i}^{2}}\delta (\omega -{\omega }_{i}\mathrm{).}$$

In the standard approach it is assumed that the initial state *ρ*(0) of the composite system *S* + *B* is uncorrelated, i.e., *ρ*(0) = *ρ*_*S*_(0) ⊗ *ρ*_*T*_(0), where *ρ*_*S*_ is an arbitrary state of the Brownian particle and *ρ*_*T*_ is an equilibrium Gibbs canonical state of thermostat of temperature *T*. Next, the thermodynamic limit is imposed meaning that the thermal reservoir is infinitely extended. Then the singular spectral function *J*(*ω*) in Eq. (5) tends to a (piecewise) continuous function.

Solving Eq. (2) for *x*(*t*) we can obtain the oscillator momentum operator *p*(*t*) from the standard relation $p(t)=M\dot{x}(t)$. It allows to calculate the mean kinetic *E*_*k*_(*t*) = 〈*p*^2^(*t*)〉/2*M* and potential ${E}_{p}(t)=M{\omega }_{0}^{2}\langle {x}^{2}(t)\rangle \mathrm{/2}$ energy of the quantum oscillator. In the long time limit, when the equilibrium state is reached, one gets the following expressions for the above quantities (see the section Methods)

6 $${E}_{k}=\mathop{lim}\limits_{t\to {\rm{\infty }}}\frac{1}{2M}\langle {p}^{2}(t)\rangle =\langle {\varepsilon }_{k}\rangle ={\int }_{0}^{{\rm{\infty }}}d\omega \,{\varepsilon }_{k}(\omega ){{\mathbb{P}}}_{k}(\omega ),$$

7 $${E}_{p}=\mathop{lim}\limits_{t\to {\rm{\infty }}}\frac{1}{2}M{\omega }_{0}^{2}\langle {x}^{2}(t)\rangle =\langle {\varepsilon }_{p}\rangle ={\int }_{0}^{{\rm{\infty }}}d\omega \,{\varepsilon }_{p}(\omega ){{\mathbb{P}}}_{p}(\omega ),$$

where

8 $${\varepsilon }_{k}(\omega )={\varepsilon }_{p}(\omega )=\frac{\hslash \omega }{4}{\rm{c}}{\rm{o}}{\rm{t}}{\rm{h}}(\frac{\hslash \omega }{2{k}_{B}T})$$

are thermally averaged kinetic and potential energies of one degree of freedom of the thermostat^23^. The latter average is over the Gibbs canonical ensemble with the statistical operator *ρ*_*T *_∝ *exp*[−*H*_*B*_/*k*_*B*_*T*], where *H*_*B*_ is the Hamiltonian of the heat bath and *k*_*B*_ is the Boltzmann constant. The probability distributions ${{\mathbb{P}}}_{k}$(*ω*) and ${{\mathbb{P}}}_{p}$(*ω*) have the form

9 $${{\mathbb{P}}}_{k}(\omega )=\frac{1}{\pi }[{\hat{R}}_{L}(i\omega )+{\hat{R}}_{L}(\,-\,i\omega )],\,\,{{\mathbb{P}}}_{p}(\omega )=\frac{iM{\omega }_{0}^{2}}{\pi \omega }[{\hat{Q}}_{L}(i\omega )-{\hat{Q}}_{L}(\,-\,i\omega )]$$

and

10 $${\hat{R}}_{L}(z)=\frac{Mz}{M{z}^{2}+z{\hat{\gamma }}_{L}(z)+M{\omega }_{0}^{2}},\,\,{\hat{Q}}_{L}(z)=\frac{1}{M{z}^{2}+z{\hat{\gamma }}_{L}(z)+M{\omega }_{0}^{2}}$$

are Laplace transforms of the response functions *R*(*t*) and *Q*(*t*) for the momentum and coordinate operator of the oscillator, respectively. The function ${\hat{\gamma }}_{L}(z)$ is the Laplace transform of the damping kernel *γ*(*t*). To be more precise, for any function *f*(*t*) its Laplace transform is defined as

11 $${\hat{f}}_{L}(z)={\int }_{0}^{\infty }\,dt\,{e}^{-zt}f(t\mathrm{).}$$

Eqs (6) and (7) are quantum counterparts of the theorem on the energy equipartition of classical systems. One can note that for quantum systems there is no equipartition but there is another form of partition of energy described by the corresponding frequency probability distributions:

(i) The mean kinetic energy *E*_*k*_ of the quantum oscillator is a thermally averaged kinetic energy per one degree of freedom of the thermostat oscillators.
(ii) The mean potential energy *E*_*p*_ of the quantum oscillator is a thermally averaged potential energy per one degree of freedom of the thermostat oscillators.

This should be contrasted with the corresponding classical system for which average energy is equally shared in the same portion *k*_*B*_*T*/2 among all degrees of freedom of the composite system, i.e.,

12 $${E}_{k}={\varepsilon }_{k}={E}_{p}={\varepsilon }_{p}={k}_{B}T\mathrm{/2.}$$

According to our above statement in the quantum case the kinetic energy is not divided equally among all degrees of freedom and thermostat oscillators contribute to *E*_*k*_ with a different degree, i.e. if the thermostat oscillator has eigenfrequency *ω* then its contribution to *E*_*k*_ is determined by the probability density ${{\mathbb{P}}}_{k}$(*ω*). Because the model is exactly solvable the probability density ${{\mathbb{P}}}_{k}$(*ω*) is exact and determined by the Laplace transform ${\hat{R}}_{L}(z)$ of the response function *R*(*t*). It contains the Laplace transform ${\hat{\gamma }}_{L}(z)$ of the memory function *γ*(*t*) which, via Eq. (3), depends on the spectral function *J*(*ω*) which in turn, via Eq. (5), comprises all information on the oscillator-thermostat interaction and frequencies of the bath modes. This argumentation applies, *mutatis mutandis*, to the mean potential energy of the oscillator.

We now consider two random variables *ξ*_*k*_ and *ξ*_*p*_ distributed according to the probability density ${{\mathbb{P}}}_{k}$(*ω*) and ${{\mathbb{P}}}_{p}$(*ω*), respectively. The first moments, i.e. the mean values 〈*ξ*_*k*_〉 and 〈*ξ*_*p*_〉 of the random variables *ξ*_*k*_ and *ξ*_*p*_ are proportional to the kinetic *E*_*k*_ and potential *E*_*p*_ energy of the oscillator at zero temperature *T* = 0, namely

13 $${E}_{k}^{0}={E}_{k}(T=0)=\frac{\hslash }{4}\langle {\xi }_{k}\rangle \,{E}_{p}^{0}={E}_{p}(T=0)=\frac{\hslash }{4}\langle {\xi }_{p}\rangle .$$

Although absolute temperature of the environment *B* is zero the central system *S* is strongly influenced by the purely quantum vacuum fluctuations of the bath and therefore its energy is always greater than zero. Hereafter, we analyse the influence of dissipation mechanisms modelled by two memory kernels, the exponentially decaying function *γ*_*D*_(*t*) (the Drude model) and the algebraically decaying one *γ*_*A*_(*t*), namely,

14 $${{\rm{\gamma }}}_{D}(t)=\frac{{{\rm{\gamma }}}_{0}}{2{\tau }_{c}}{e}^{-t/{\tau }_{c}},\,\,{\gamma }_{A}(t)=\frac{{{\rm{\gamma }}}_{0}}{\pi }\,\frac{{\tau }_{c}}{{t}^{2}+{\tau }_{c}^{2}}\mathrm{.}$$

The corresponding form of the spectral density *J*(*ω*) is obtained from Eqs (3) and (27):

15 $${J}_{D}(\omega )=\frac{1}{\pi }\frac{{{\rm{\gamma }}}_{0}}{1+{\omega }^{2}{\tau }_{c}^{2}},\,\,{J}_{A}(\omega )=\frac{{{\rm{\gamma }}}_{0}}{\pi }{e}^{-\omega {\tau }_{c}}\mathrm{.}$$

In the above scaling, if the memory time *τ*_*c*_ → 0, both functions γ_*D*_(*t*) and γ_*A*_(*t*) tend to the Dirac delta and the integral term in the GQLE (2) reduces to the frictional force of the Stokes form. For classical systems it corresponds to the limit of Gaussian white noise when thermal noise is *δ*-correlated. There are four parameters: *M*, γ_0_, *τ*_*c*_ and *ω*_0_ and three characteristic times (or frequencies being their reciprocals): *τ*_*v*_ = *M*/γ_0_, *τ*_*c*_, 1/*ω*_0_. If we rescale all quantities to the dimensionless form then there are only two dimensionless parameters

16 $$\alpha =\frac{M}{{\tau }_{c}{{\rm{\gamma }}}_{0}}=\frac{{\tau }_{v}}{{\tau }_{c}},\,\,{\tilde{\omega }}_{0}={\omega }_{0}{\tau }_{v},$$

where *α* is a ratio of two characteristic times *τ*_*v*_ and *τ*_*c*_. There is an alternative scaling with ${\hat{\omega }}_{0}={\omega }_{0}{\tau }_{c}$ but since we will be interested mainly in the role of the memory time we use only (16). We would like to pay attention that in this scaling the parameter *τ*_*v*_ is fixed and the change of *α* means the change of the memory time *τ*_*c*_.

### Mean kinetic and potential energy

First, we consider the Drude model for which

17 $${{\mathbb{P}}}_{k}(\omega )=\frac{2}{\pi }\frac{2{{\rm{\gamma }}}_{0}M{\omega }^{2}}{{\omega }^{2}{[{{\rm{\gamma }}}_{0}+2M{\tau }_{c}({\omega }_{0}^{2}-{\omega }^{2})]}^{2}+4{M}^{2}{({\omega }^{2}-{\omega }_{0}^{2})}^{2}}\mathrm{.}$$

In the case of algebraic decay of γ(*t*) as it is in Eq. (14), it takes the form

18 $${{\mathbb{P}}}_{k}(\omega )=\frac{4\pi {{\rm{\gamma }}}_{0}M{\omega }^{2}{e}^{-\omega {\tau }_{c}}}{{C}_{1}(M,{{\rm{\gamma }}}_{0},{\tau }_{c},{\omega }_{0},\omega ){C}_{2}(M,{{\rm{\gamma }}}_{0},{\tau }_{c},{\omega }_{0},\omega )}$$

with

$$\begin{array}{ccc}{C}_{1}(M,{\gamma }_{0},{\tau }_{c},{\omega }_{0},\omega ) & = & 2\pi M({\omega }^{2}-{\omega }_{0}^{2})+2{\gamma }_{0}\,\omega \,Ci\,(i\omega {\tau }_{c})\,\sinh (\omega {\tau }_{c})\\  & + & {\gamma }_{0}\,\omega \,\cosh (\omega {\tau }_{c})(\,-\,i\pi -2\,{\rm{S}}{\rm{h}}{\rm{i}}\,(\omega {\tau }_{c})),\end{array}$$

$$\begin{array}{ccc}{C}_{2}(M,{\gamma }_{0},{\tau }_{c},{\omega }_{0},\omega ) & = & 2\pi M({\omega }^{2}-{\omega }_{0}^{2})+2{\gamma }_{0}\,\omega \,{\rm{C}}{\rm{i}}\,(\,-\,i\omega {\tau }_{c})\,\sinh (\omega {\tau }_{c})\\  & + & i{\gamma }_{0}\,\omega \,\cosh (\omega {\tau }_{c})(\pi +2i\,{\rm{S}}{\rm{h}}{\rm{i}}\,(\omega {\tau }_{c}))\end{array}$$

and

19 $$Ci\,(z)=-\,{\int }_{z}^{{\rm{\infty }}}dt\,\frac{\cos t}{t},\,\,{\rm{S}}{\rm{h}}{\rm{i}}\,(z)={\int }_{0}^{z}dt\,\frac{\sinh t}{t}.$$

The expressions for the corresponding ${{\mathbb{P}}}_{p}$(*ω*) can be obtained from Eq. (17) or (18) by changing ${\omega }^{2}\to {\omega }_{0}^{2}$ in their numerators. In all figures, we use dimensionless quantities and parameters. In particular, the rescaled probability densities ${\tilde{{\mathbb{P}}}}_{k}(x)=\mathrm{(1/}{\tau }_{v}){{\mathbb{P}}}_{k}(x/{\tau }_{v})$ and ${\tilde{{\mathbb{P}}}}_{p}(x)=\mathrm{(1/}{\tau }_{v}){{\mathbb{P}}}_{p}(x/{\tau }_{v})$, where *x* = *ωτ*_*v*_ is a dimensionless frequency and *τ*_*v*_ is fixed. In consequence, the change of the parameter *α* = *τ*_*v*_/*τ*_*c*_ denotes the change of the memory time *τ*_*c*_.

**Figure 1:**
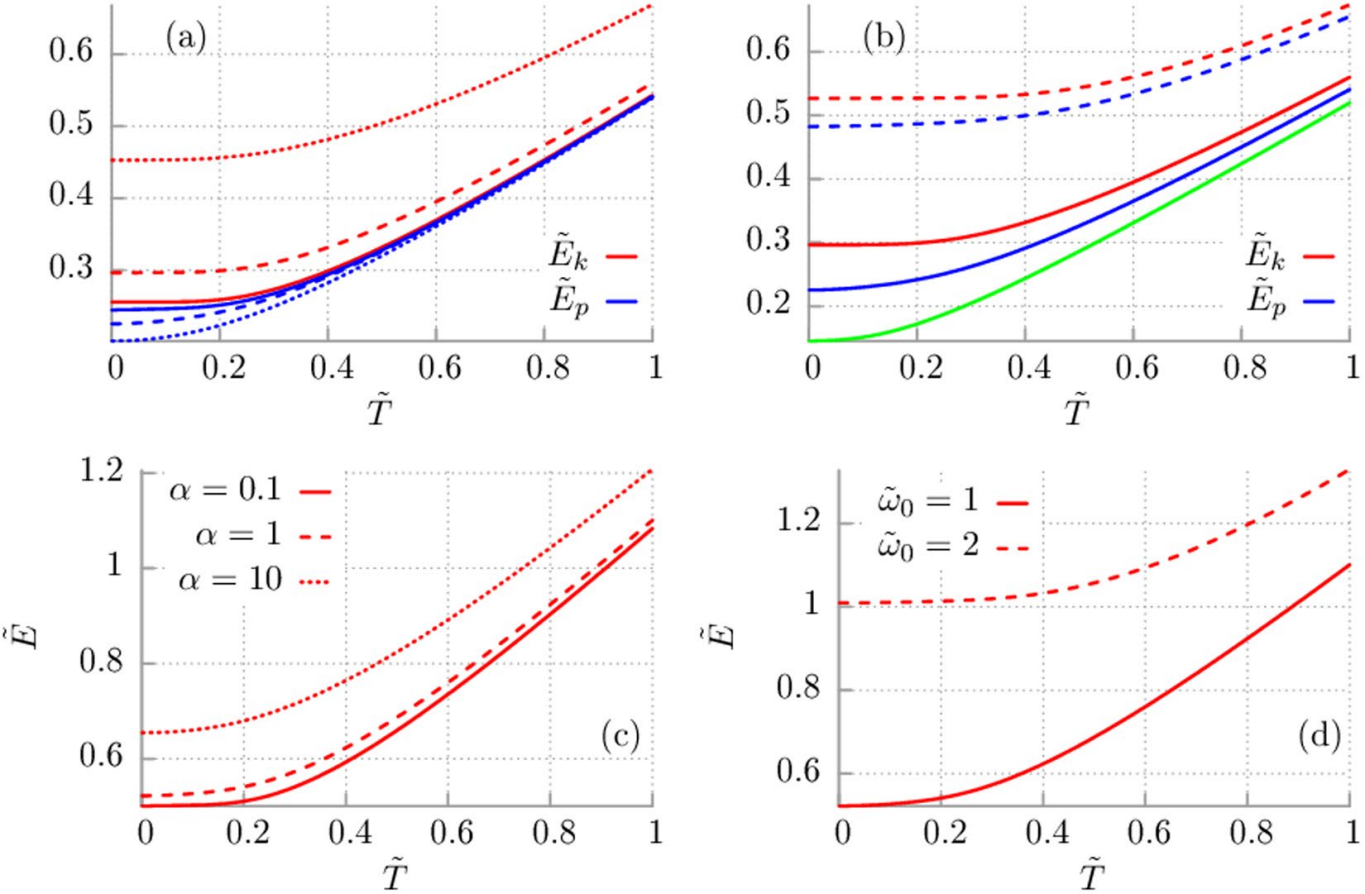
Drude model of dissipation. The dimensionless mean kinetic energy ${\tilde{E}}_{k}={\tau }_{v}{E}_{k}/\hslash$ (red) and mean potential energy ${\tilde{E}}_{p}={\tau }_{v}{E}_{p}/\hslash$ (blue) versus dimensionless temperature $\tilde{T}={\tau }_{v}{k}_{B}T/\hslash$, where ${\tau }_{v}=M/{\gamma }_{0}$ is fixed. Panel (a): Solid line $\alpha ={\tau }_{v}/{\tau }_{c}\mathrm{=0.1}$, dashed line: $\alpha \mathrm{=1}$, dotted line $\alpha \mathrm{=10}$; all for the fixed eigenfrequency ${\tilde{\omega }}_{0}={\omega }_{0}{\tau }_{v}\mathrm{=1}$. Panel (b): Solid line ${\tilde{\omega }}_{0}\mathrm{=1}$, dashed line ${\tilde{\omega }}_{0}\mathrm{=2}$ and fixed $\alpha \mathrm{=1}$. The exception here is the green solid line which shows the mean kinetic energy ${\tilde{E}}_{k}$ for the free Brownian particle with ${\tilde{\omega }}_{0}\mathrm{=0}$. Panel (c): The total energy $\tilde{E}={\tilde{E}}_{k}+{\tilde{E}}_{p}$ corresponding to the regime of panel (a). Panel (d): The total energy corresponding to the regime of panel (b).

In Fig. 1 we illustrate the mean kinetic and potential energy determined by Eq. (6) and (7), respectively, as a function of temperature for selected values of the model parameters. In particular, in panel (a) we present the influence of the memory time *τ*_*c*_ via the parameter *α* = *τ*_*v*_/*τ*_*c*_ with fixed *τ*_*v*_ = *M*/*γ*_0_ and the oscillator eigenfrequency ${\tilde{\omega }}_{0}=1$. We note that regardless of the value of the memory time for this set of parameters the potential energy is always smaller than the kinetic one. Moreover, when the memory time decreases (i.e. *α* increases) the kinetic energy increases whereas the potential one is decreasing. On the other hand if time *τ*_*c*_ increases (i.e. *α* decreases) then the difference between the kinetic and potential energy is getting smaller and smaller and in the limit of infinitely long memory time it tends to zero. Alternatively, if the memory time *τ*_*c*_ is fixed and we change *τ*_*v*_ = *M*/*γ*_0_ in *α* = *τ*_*v*_/*τ*_*c*_ we observe that the kinetic and potential energy approaches the same value in the limit of large values of *α* (not depicted). It implies that either (i) the mass *M* of the particle is large or (ii) the coupling *γ*_0_ between the system and thermostat is weak. In the latter situation one could say that the system may be approximated by a free harmonic oscillator, which especially in the low temperature limit approaches a coherent state, where the position and momentum variances (proportional to kinetic and potential energy) match. The problem of relation between the kinetic and potential energy is discussed also in ref.^19^.

In panel (b) of Fig. 1 we present the same characteristics but now depicted for the fixed memory time *α* = 1 and different values of the oscillator eigenfrequency ${\tilde{\omega }}_{0}$. The observation is that for increasing values of the latter parameter both the kinetic and potential energy is growing. However, still the kinetic one is larger than the potential energy. The reader should note there also the interesting comparison with the case of a free quantum Brownian particle ${\tilde{\omega }}_{0}\mathrm{=0}$ which is marked by the green colour. It turns out that the kinetic energy of a quantum harmonic oscillator is always greater than in the corresponding case of the free particle.

In panels (c) and (d) of Fig. 1 we analyse the dependence of the total averaged energy $\tilde{E}={\tilde{E}}_{k}+{\tilde{E}}_{p}$ of the quantum oscillator versus the previously discussed parameters. It is instructive to observe in the panel (c) that when the memory time *τ*_*c*_ decreases (i.e. *α* increases) the total energy of the system increases to infinity. It means that the limiting case of vanishing memory is non-physical for quantum systems. Since the time scale *τ*_*c*_ can be viewed also as the leading correlation time of the quantum thermal fluctuations one would say in analogy to classical physics that there is no limit of white noise in the quantum realm. In other words it implies that quantum thermal fluctuations are always correlated. Qualitatively, the dependence of the kinetic, potential or total energy on temperature is robust with respect to changes of the model parameter values. For high enough temperature it always tends to the classical limit *k*_*B*_*T*/2 while in the regime of low temperature it is higher than the corresponding classical value. Note that all curves are monotonically increasing functions of temperature which never intersect each other. Due to this fact for a qualitative analysis it is sufficient to study the oscillator energies corresponding to zero temperature limit *T* = 0.

Here, we mention two recent papers^24,25^ where similar problems are studied. There the variance of the position of the quantum Brownian particle is studied as a function of temperature and the system-thermostat coupling strength. One of the main results of analysis performed there is the particle position squeezing as temperature decreases and the interaction strength increases. For our system we observe a similar effect (not depicted). The potential energy *E*_*p*_ (the particle position variance) decreases for fixed temperature *T* and growing of the coupling constant *γ*_0_. It then translates to the fact that the probability distribution ${{\mathbb{P}}}_{p}$(*ω*) corresponding to the mean potential energy rapidly decays meaning that relatively only the oscillators of low frequency bring the contribution to the average potential energy. Under this assumption they have small kinetic energy and therefore can transfer only little amount of it to the system. Consequently, the variance of the particle position is limited. In contrast, for weak system-thermostat coupling oscillators of high frequency dominate the probability distribution for the potential energy (position variance). Then they are allowed to have much larger kinetic energy and may transfer much bigger portion of it to the system resulting in increase of the particle position variance. Therefore the theorem of quantum partition of energy turns out to be quite helpful in qualitative interpretation of the mentioned particle position squeezing effect.

**Figure 2:**
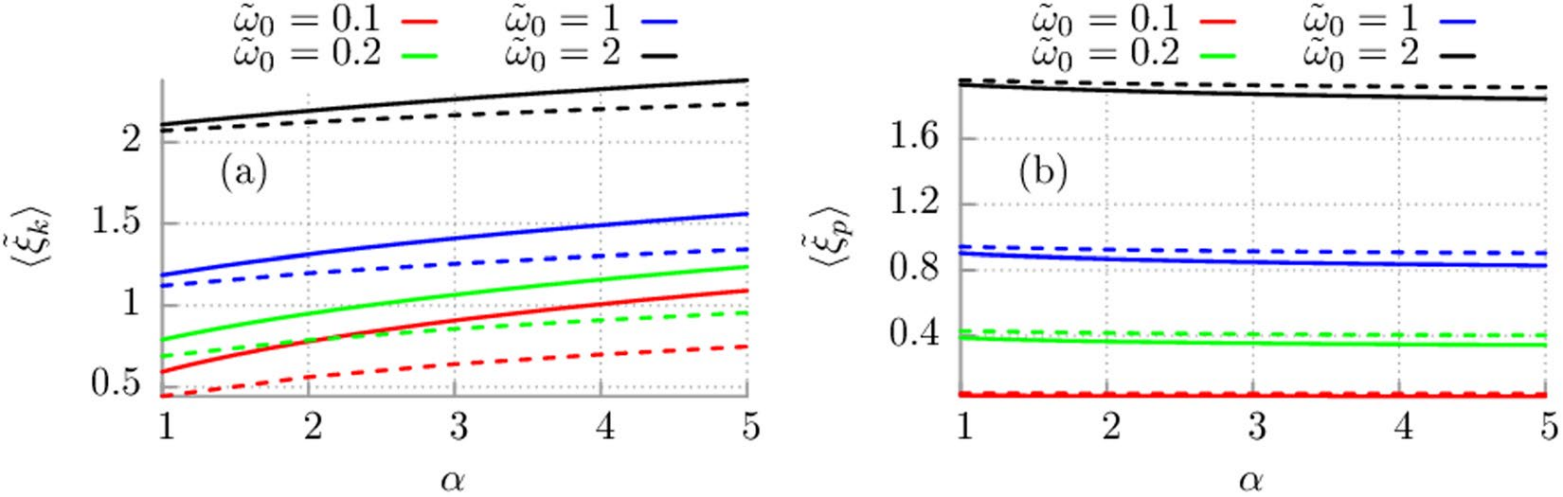
Panel (a): the mean value $\langle {\tilde{\xi }}_{k}\rangle ={\tau }_{v}\langle {\xi }_{k}\rangle$ of the random variable distributed according to the probability distribution ${\tilde{{\mathbb{P}}}}_{k}(x)=\mathrm{(1/}{\tau }_{v}){{\mathbb{P}}}_{k}(x/{\tau }_{v})$ corresponding to the mean kinetic energy of the quantum harmonic oscillator is shown as the function of the parameter $\alpha ={\tau }_{v}/{\tau }_{c}$, where ${\tau }_{v}=M/{{\rm{\gamma }}}_{0}$ is fixed, and different values of eigenfrequency ${\tilde{\omega }}_{0}={\omega }_{0}{\tau }_{v}$. Panel (b): the first statistical moment of the probability density ${\tilde{{\mathbb{P}}}}_{p}(x)=\mathrm{(1/}{\tau }_{v}){{\mathbb{P}}}_{p}(x/{\tau }_{v})$ for the potential energy of the quantum harmonic oscillator. Solid lines correspond to the Drude (exponential) model and dashed lines to algebraic decay of $\gamma (t)$.

The case of zero temperature *T* = 0 is analysed in Fig. 2 where the impact of the memory time *τ*_*c*_ as well as the eigenfrequency ${\tilde{\omega }}_{0}$ is shown. Now additionally we compare the two mentioned mechanisms of dissipation. Panel (a) of this figure shows that when the memory time *τ*_*c*_ decreases (i.e. *α* increases) the kinetic energy monotonically increases. The opposite effect is for the potential energy: it slowly decreases as the memory time is shorter. One can note that kinetic energy for Drude model is greater than for the algebraic decay of γ(*t*). For the potential energy it is opposite sequence: *E*_*p*_ is greater for the algebraic decay of γ than for the exponential one. Moreover, both the kinetic as well as potential energy grows as the eigenfrequency ${\tilde{\omega }}_{0}$ is increased. Finally, the influence of the coupling strength γ_0_ should be pointed out (not shown in figures). It seems to be rather obvious that if the coupling is stronger then more channels are open to transmit energy from environment to the central system *S* and therefore its energy is greater.

### Information provided in probability distributions

In reach literature, formulas for the average kinetic and/or potential energy of the dissipative quantum harmonic oscillator appear in various context in original papers and textbooks. We can mention several of them: Eq. (83) in ref.^26^, Table 2 of ref.^17^, Eq. (4.14) in ref.^27^ or Eq. (3.475) in ref.^28^. The expressions for the mean kinetic and/or potential energy can also be obtained directly or indirectly from different forms of fluctuation-dissipation relations^29–32^ which are derived in the framework of the linear response theory which relates relaxation of a weakly perturbed system to the spontaneous fluctuations in thermal equilibrium, see e.g. Eq. (6.85) and (6.87) in ref.^1^ and Eq. (3.498) and (3.499) in ref.^28^. Therefore although the calculation of both kinetic and potential energy for a dissipative quantum oscillator has been done, the interpretation of these results as a quantum counterpart of the equipartition theorem expressed by the probability distributions ${{\mathbb{P}}}_{k}$(*x*) or ${{\mathbb{P}}}_{p}$(*x*) represents, to the best of our knowledge, an original point of view which may help to improve the general understanding of the physics of dissipative quantum systems.

**Figure 3:**
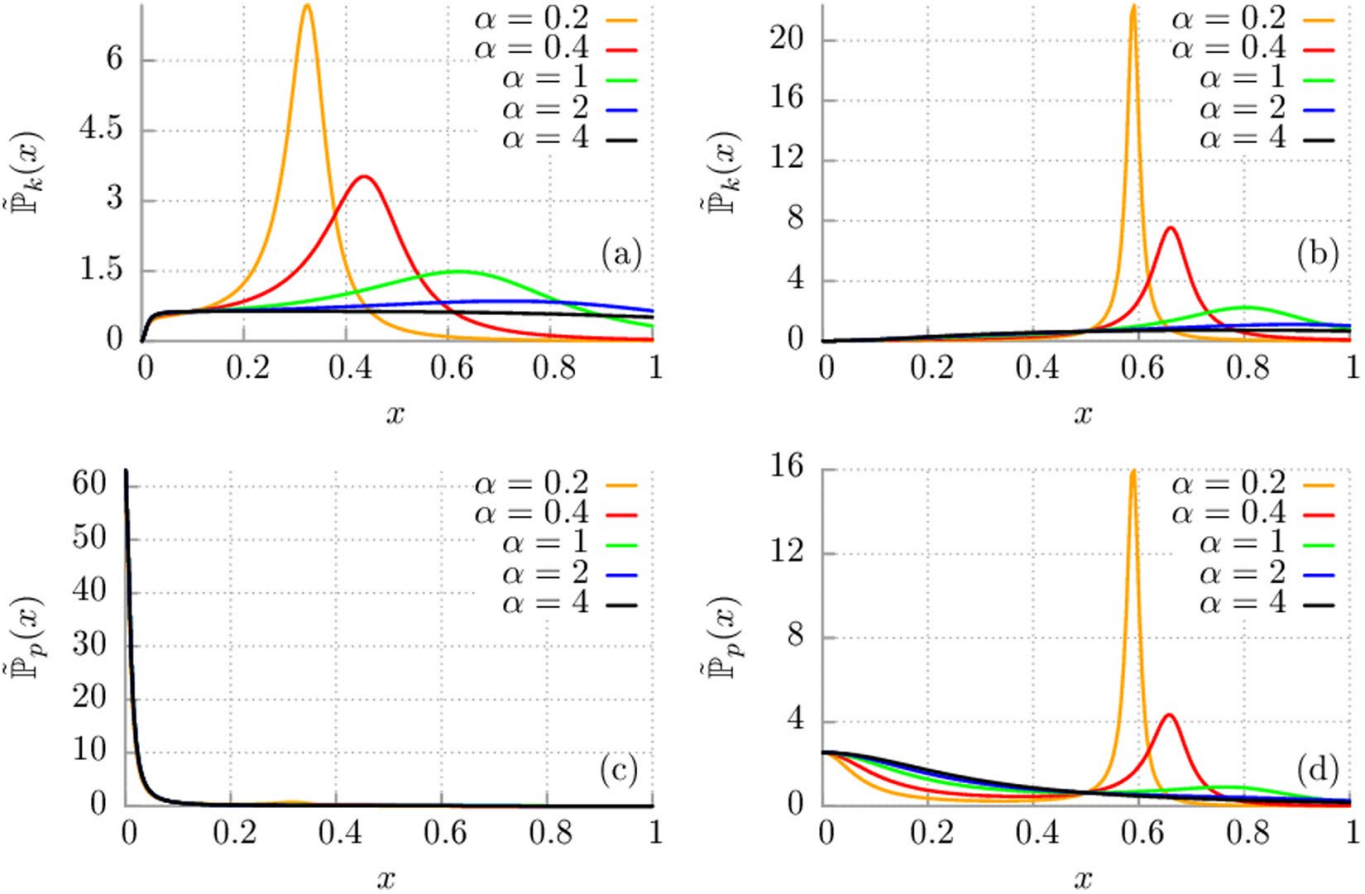
Drude model of dissipation. The probability distribution ${\tilde{{\mathbb{P}}}}_{k}(x)=\mathrm{(1/}{\tau }_{v}){{\mathbb{P}}}_{k}(x/{\tau }_{v})$ corresponding to the mean kinetic energy of the quantum harmonic oscillator is depicted for selected values of the parameter $\alpha ={\tau }_{v}/{\tau }_{c}$ with fixed ${\tau }_{v}=M/{\gamma }_{0}$ and ${\tilde{\omega }}_{0}\mathrm{=0.1}$ [panel (a)] and ${\tilde{\omega }}_{0}\mathrm{=0.5}$ [panel (b)]. Panel (c) and (d): The probability density ${\tilde{{\mathbb{P}}}}_{p}(x)$ corresponding to the mean potential energy of the quantum harmonic oscillator is shown for different $\alpha$, fixed ${\tilde{\omega }}_{0}\mathrm{=0.1}$ and ${\tilde{\omega }}_{0}\mathrm{=0.5}$, respectively.

In Fig. 3 we depict the dimensionless probability distribution ${\tilde{{\mathbb{P}}}}_{k}(x)$ for the Drude dissipation mechanism and selected values of the model parameters. The general observation is that the thermostat oscillators contribute to the energetics of the central system in a noticeably different way. In panel (a) and (b) we present the probability distribution ${\tilde{{\mathbb{P}}}}_{k}(x)$ corresponding to the kinetic energy ${\tilde{E}}_{k}$ of the oscillator. The reader can observe that if the memory time *τ*_*c*_ is large (i.e. *α* is small) then the probability distribution is peaked around some optimal frequency *x*_*m*_ which brings the greatest contribution to the energy of system. On the other hand, if the memory time is getting smaller then the probability distribution is progressively flattened. The influence of the oscillator eigenfrequency ${\tilde{\omega }}_{0}$ is depicted in panel (b). We note that an increase of this parameter causes shifting of the density towards larger frequencies *x*. However, in each case the overall shape is conserved. A radically different behaviour is observed for the distribution ${\tilde{{\mathbb{P}}}}_{p}(x)$ corresponding to the potential energy ${\tilde{E}}_{p}$ of the oscillator. We illustrate it in the panel (c) and (d) of the same figure. In particular, we note that when the eigenfrequency ${\tilde{\omega }}_{0}$ of the system is small this distribution is robust with respect to changes of the memory time *α*, c.f. panel (c). Then it is a rapidly decreasing function of the frequency which means that only thermostat oscillators of very small frequency contribute significantly to the potential energy of the system. It is distinctly different than it was in the case of the probability distribution for the kinetic energy. The situation changes for larger values of the oscillator eigenfrequency ${\tilde{\omega }}_{0}$, c.f. panel (d). Then for the long enough memory time (small *α*) these two densities start to resemble each other. It means that both distributions are peaked and only thermostat oscillators taken from a very narrow interval of frequencies contribute to the corresponding energy of the system.

**Figure 4:**
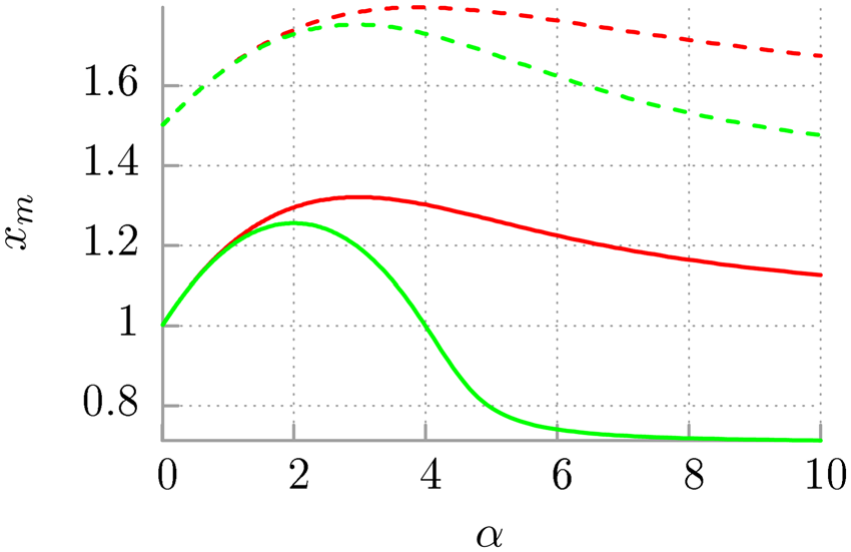
The dependence of the optimal frequency ${x}_{m}$ of the thermostat oscillators (at which the probability distribution is maximal) on the parameter $\alpha$ for the Drude dissipation mechanism. The red and green colour corresponds to the kinetic ${\tilde{E}}_{k}$ and potential ${\tilde{E}}_{p}$ energy. The solid and dashed lines are for ${\tilde{\omega }}_{0}=1$ and ${\tilde{\omega }}_{0}=1.5$, respectively.

In Fig. 4 we present the dependence of the optimal frequency *x*_*m*_ of the thermostat oscillators (at *x*_*m*_ the probability distribution has maximum) upon the parameter *α* for the Drude dissipation mechanism and selected values of the eigenfrequency ${\tilde{\omega }}_{0}$. For fixed values of *τ*_*v*_ = *M*/*γ*_0_ and *ω*_0_ the function *x*_*m*_(*α*) displays a non-monotonic character for the kinetic as well as the potential energy. It means that there is a singled out value of *α* (i.e. the memory time *τ*_*c*_) for which *x*_*m*_ is maximal. We should stress that *x*_*m*_ for the kinetic energy is greater than *x*_*m*_ for the potential energy. We can also conclude that for large *α* oscillators of relatively lower frequency dominate in the contribution to both kinetic and potential energy. In this panel we also depict the impact of the eigenfrequency ${\tilde{\omega }}_{0}$ on this characteristic. An increase of the latter parameter causes shifting of the curve towards larger values of *x*_*m*_, however, the overall shape of the functional dependence remains unchanged.

**Figure 5:**
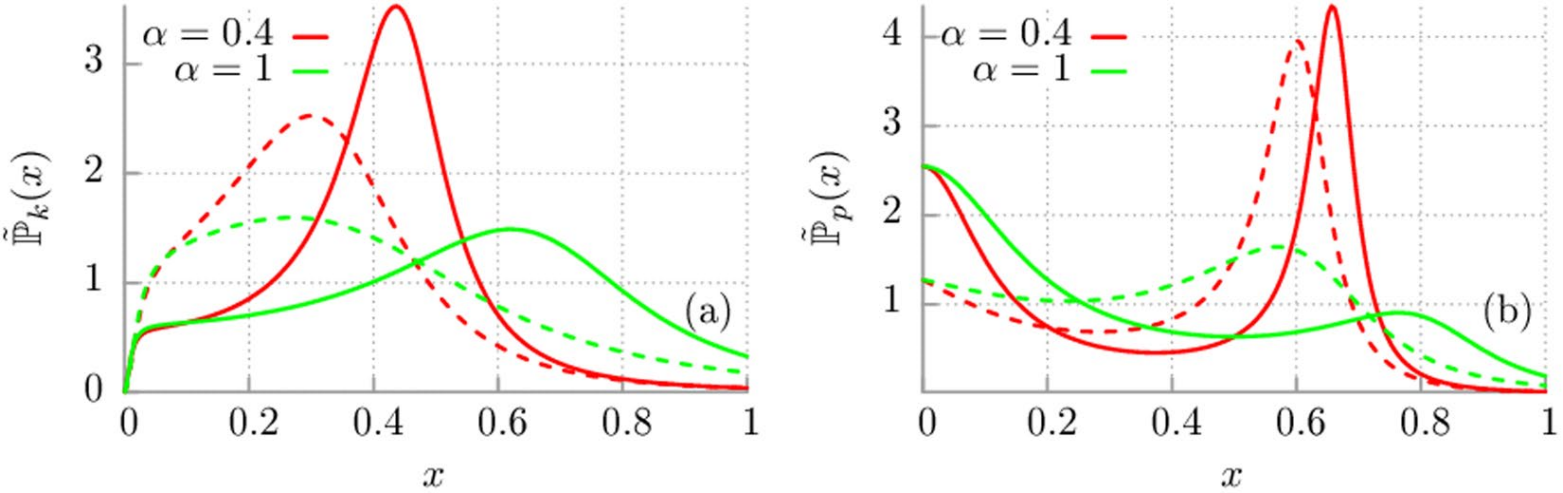
Comparison of the impact of Drude (solid lines) and algebraic (dashed lines) decay of the memory function. Panel (a): The probability distribution corresponding to the mean kinetic energy of the quantum harmonic oscillator is depicted for two values of $\alpha$ and for the frequency ${\tilde{\omega }}_{0}\mathrm{=0.1}$. Panel (b): The probability density ${\tilde{{\mathbb{P}}}}_{p}(x)$ corresponding to the mean potential energy of the quantum harmonic oscillator is shown for different magnitudes of the parameter $\alpha$ and ${\tilde{\omega }}_{0}\mathrm{=0.5}$.

Last but not least, in Fig. 5 we compare the probability distributions ${\tilde{{\mathbb{P}}}}_{k}(x)$ and ${\tilde{{\mathbb{P}}}}_{p}(x)$ for the both considered dissipation mechanisms, i.e. exponential (Drude) and algebraic. The general remark is that the shape of the distributions ${\tilde{{\mathbb{P}}}}_{k}(x)$ and ${\tilde{{\mathbb{P}}}}_{p}(x)$ are qualitatively similar for the exponential and algebraic memory kernel. The difference is only visible in quantitative way. The important thing to note is that regardless of the values of the oscillator eigenfrequency ${\tilde{\omega }}_{0}$ the optimal frequencies which brings the most pronounced contribution to the kinetic as well as potential energy in the Drude model are higher than for the corresponding ones in the case of algebraic dissipation.

## Discussion

We analysed partition of energy of the dissipative quantum harmonic oscillator. Mean kinetic and potential energy of the system are mean kinetic and potential energies of the thermostat per one degree of freedom, i.e., ${E}_{k}=\langle {\varepsilon }_{k}\rangle$ and ${E}_{p}=\langle {\varepsilon }_{p}\rangle$, where $\langle {\varepsilon }_{k}\rangle$ and $\langle {\varepsilon }_{p}\rangle$ are mean kinetic and potential energies per one degree of freedom of the thermostat which consists of harmonic oscillators too. The symbol 〈...〉 denotes two-fold averaging: (i) over the Gibbs canonical state for the thermostat and (ii) over thermostat oscillators frequencies *ω* which contribute to *E*_*k*_ and *E*_*p*_ according to the probability distribution ${{\mathbb{P}}}_{k}$(*ω*) and ${{\mathbb{P}}}_{p}$(*ω*), respectively. The relation for kinetic energy partition is similar to that for classical systems: The mean kinetic energy of the oscillator equals the mean kinetic energy of the thermostat degree of freedom. Of course, for classical systems the mean value of kinetic energy is *k*_*B*_*T*/2 and depends only on temperature of thermostat. In the quantum case, it depends on “everything” (system-thermostat coupling, memory time, temperature).

We considered two examples of the dissipation mechanism: the Drude model characterised by the exponentially decreasing function and the algebraic decay of the memory kernel. We compared them and conclude that in the case of Drude model the kinetic energy of the oscillator is greater than for the algebraic decay. On the other hand, the reversed scenario is observed for the potential energy where the algebraic decay dominates the Drude dissipation. Moreover, the probability distributions are similar in both cases and display only quantitative differences. For the Drude model the optimal frequencies of thermostat oscillators which bring the largest contribution to the kinetic and potential energy are slightly higher than for the algebraic decay.

We have to emphasize that the quantum system which is open but not dissipative, does not obey the relations (6) and (7) for the energy partition. What we need is the thermodynamic limit for thermostat. The system is open when it interacts with environment but if the environment is a system of finite degrees of freedom then the memory function and the correlation function of quantum noise are quasi-periodic functions of time and the thermodynamic equilibrium state cannot be reached. In the case of finite thermostat, the response function *R*(*t*) in Eq. (25) is quasi-periodic, all three terms contribute to 〈*p*^2^(*t*)〉 and the limit *t* → ∞ does not exist for 〈*p*^2^(*t*)〉.

One more issue should be discussed. When the memory time *τ*_*c*_ tends to zero, then the average energy of the oscillator increases to infinity. On the other hand, when *τ*_*c*_ → 0, the spectral density *J*(*ω*) is constant (the ohmic dissipation), the memory function γ(*t*) → *δ*(*t*), the integro-differential Langevin equation becomes local in time (as for classical Markovian processes) and is similar to a classical Newton equation with noise. However, we should also consider the correlation function *C*(*t*) of noise *η*(*t*). From Eq. (26) it follows that when *J*(*ω*) is constant then

20 $$C(t)\propto {\int }_{0}^{{\rm{\infty }}}\,d\omega \,\frac{\hslash \omega }{2}\,{\rm{c}}{\rm{o}}{\rm{t}}{\rm{h}}(\frac{\hslash \omega }{2{k}_{B}T})\cos \,(\omega t).$$

We see that it does not tend to white noise as in the classical case. It is even worse: it diverges! We refer the interested reader to ref.^33^ for a more detailed analysis and to ref.^17^ for discussion on the ohmic dissipation and Markovian limit. Another aspect of the short memory time limit has been discussed for dynamics of solitons in superfluids^34^. This formal limit and the corresponding Markovian approximation gives rise to the Abraham-Lorentz force (i.e., a term proportional to the derivative of the soliton’s acceleration) which results in breaking of causality. Three above non-physical effects lead to the conclusion that the limiting case of vanishing memory time is not allowable for quantum systems.

By the paradigmatic example of a quantum harmonic oscillator we demonstrated the quantum counterpart of the energy equipartition theorem which holds for classical systems. It is conceptually simple yet powerful tool for analysis of quantum open systems. Therefore we hope that our work in near future will open a new avenues within the area of physics.

## Methods

### Solutions of the generalized quantum Langevin equation

In this section we present details of derivation of expressions for the averaged kinetic and potential energies of the quantum oscillator, i.e. Eqs (6) and (7). The integral kernel of the GQLE (2) is of convolution type and applying the Laplace transform yields the algebraic form,

21 $${\hat{x}}_{L}(z)={\hat{R}}_{L}(z)x\mathrm{(0)}+{\hat{Q}}_{L}(z)p\mathrm{(0)}+{\hat{Q}}_{L}(z){\hat{\eta }}_{L}(z),$$

where ${\hat{R}}_{L}(z)$ and ${\hat{Q}}_{L}(z)$ are defined in Eq. (10). The inverse Laplace transform of Eq. (21) gives the solution

22 $$x(t)=R(t)x\mathrm{(0)}+Q(t)p\mathrm{(0)}+{\int }_{0}^{t}\,Q(t-s)\,\eta (s)ds\mathrm{.}$$

From the theory of Laplace transform it follows that ${lim}_{z\to \infty }\,\hat{f}(z)=0$ for any function *f*(*t*) for which the Laplace transform exists. In particular, it is also true for the functions ${f}_{1}(t)=\dot{R}(t)=dR(t)/dt$ and ${f}_{2}(t)=\dot{Q}(t)$. Calculating their Laplace transform, we obtain the relations

23 $$R\mathrm{(0)}=\mathop{lim}\limits_{z\to \infty }z{\hat{R}}_{L}(z)=\mathrm{1,}\,\,Q\mathrm{(0)}=\mathop{lim}\limits_{z\to \infty }z{\hat{Q}}_{L}(z)=\mathrm{0,}$$

which of course should be satisfied because of (22) for *t* = 0. To derive a solution for the momentum *p*(*t*) we note that the Laplace transform of the velocity is ${\hat{v}}_{L}(z)=z{\hat{x}}_{L}(z)-x\mathrm{(0)}$ and for the momentum one gets ${\hat{p}}_{L}(z)=Mz{\hat{x}}_{L}(z)-Mx\mathrm{(0)}$. We insert ${\hat{x}}_{L}(z)$ from Eq. (21) and utilize the equality for the Laplace transform of derivative of the function $\dot{R}(t)$, i.e., ${\hat{\dot{R}}}_{L}(z)=z{\hat{R}}_{L}(z)-1$. The result is

24 $${\hat{p}}_{L}(z)=M{\hat{R}}_{L}(z)x\mathrm{(0)}+{\hat{Q}}_{L}(z)p\mathrm{(0)}+{\hat{Q}}_{L}(z){\hat{\eta }}_{L}(z\mathrm{).}$$

Its inverse Laplace transform yields the solution

25 $$p(t)=R(t)p\mathrm{(0)}+M\dot{R}(t)x\mathrm{(0)}+{\int }_{0}^{t}\,R(t-s)\eta (s)ds,$$

Applying the similar method as above Eq. (24), one can show that $\dot{R}\mathrm{(0)}=0$

### Fluctuation-dissipation relation

Quantum noise *η*(*t*) defined by Eq. (4) is a family of non-commuting operators whose commutators are *c*-numbers. Its mean value over the Gibbs canonical state is zero, 〈*η*(*t*)〉 ≡ Tr[*η*(*t*)*ρ*_*T*_] = 0 and the symmetrized correlation function *C*(*t*_1_,*t*_2_) = (1/2)〈*η*(*t*_1_)*η*(*t*_2_) + *η*(*t*_2_)*η*(*t*_1_)〉 ≡ 〈[*η*(*t*_1_);*η*(*t*_2_)]_+_〉 depends on the time difference, *C*(*t*_1_,*t*_2_) = *C*(*t*_1_ − *t*_2_) = *C*(*τ*). For *τ* = *t*_1_ − *t*_2_ it takes the form:

26 $$C(\tau )=\sum _{i}\,\frac{\hslash {c}_{i}^{2}}{2{m}_{i}{\omega }_{i}}coth(\frac{\hslash {\omega }_{i}}{2{k}_{B}T})\cos ({\omega }_{i}\tau )={\int }_{0}^{{\rm{\infty }}}\,d\omega \,\frac{\hslash \omega }{2}{\rm{c}}{\rm{o}}{\rm{t}}{\rm{h}}(\frac{\hslash \omega }{2{k}_{B}T})J(\omega )\cos (\omega \tau ),$$

where the spectral density *J*(*ω*) is defined in Eq. (5). For an even function *f*(*t*), we define the pair of Fourier cosine transforms by the relations

27 $$f(t)={\int }_{0}^{{\rm{\infty }}}\,d\omega \,{\hat{f}}_{F}(\omega )\cos (\omega t),\,\,{\hat{f}}_{F}(\omega )=(2/\pi ){\int }_{0}^{{\rm{\infty }}}\,dt\,f(t)\cos (\omega t).$$

We introduce the Fourier cosine transforms of the dissipation ${\hat{{\rm{\gamma }}}}_{F}(\omega )$ and correlation ${\hat{C}}_{F}(\omega )$ functions and compare them with Eq. (3). One notice that the following equality

28 $${\hat{C}}_{F}(\omega )=\frac{\hslash \omega }{2}\,\coth (\frac{\hslash \omega }{2{k}_{B}T}){\hat{{\rm{\gamma }}}}_{F}(\omega )$$

is satisfied. It is one of the form of the fluctuation-dissipation theorem^22,29–31^ (the extended discussion on this subject is also in Chapter 3 of ref.^32^). It relates the memory kernel γ(*t*) to the correlation function of the quantum thermostat noise *η*(*t*) via its Fourier cosine transforms. On the other hand, ${\hat{C}}_{F}(\omega )$ is the Fourier transform of the correlation function *C*(*t*) of the noise *η*(*t*) and it is also called the power spectrum of noise.

### Potential energy in an equilibrium state

We calculate averaged potential energy of the quantum harmonic oscillator in the long time limit *t* → ∞ when a thermal equilibrium state is reached. From Eq. (22) we can obtain the symmetrized position-position correlation function 〈[*x*(*t*);*x*(*s*)]_+_〉. For enough long times, i.e. much longer than the characteristic time scales *τ*_*v*_ = *M*/γ_0_, *τ*_*c*_ and 1/*ω*_0_ only the last term of (22) contributes and then

29 $$\langle {[x(t);x(s)]}_{+}\rangle ={\int }_{0}^{t}\,d{t}_{1}{\int }_{0}^{s}\,d{t}_{2}\,Q(t-{t}_{1})Q(s-{t}_{2})\langle {[\eta ({t}_{1});\eta ({t}_{2})]}_{+}\rangle \mathrm{.}$$

Now, we express the correlation function *C*(*t*_1_ − *t*_2_) = 〈[*η*(*t*_1_);*η*(*t*_2_)]_+_〉 by its Fourier cosine transform to get

30 $$\langle {[x(t);x(s)]}_{+}\rangle ={\int }_{0}^{{\rm{\infty }}}\,d\omega \,{\hat{C}}_{F}(\omega ){\int }_{0}^{t}\,d{t}_{1}{\int }_{0}^{s}\,d{t}_{2}\,Q(t-{t}_{1})Q(s-{t}_{2})\cos [\omega ({t}_{1}-{t}_{2})].$$

In particular, for *t* = *s*, it is the second statistical moment of the position operator,

31 $$\langle {x}^{2}(t)\rangle ={\int }_{0}^{{\rm{\infty }}}\,d\omega \,{\hat{C}}_{F}(\omega ){\int }_{0}^{t}\,d{t}_{1}{\int }_{0}^{t}\,d{t}_{2}\,Q(t-{t}_{1})Q(t-{t}_{2})\cos [\omega ({t}_{1}-{t}_{2})].$$

We introduce new integration variables *τ* = *t* − *t*_1_ and *u* = *t* − *t*_2_ and convert equation (31) into the form

32 $$\langle {x}^{2}(t)\rangle ={\int }_{0}^{{\rm{\infty }}}\,d\omega \,{\hat{C}}_{F}(\omega ){\int }_{0}^{t}\,d\tau {\int }_{0}^{t}\,du\,Q(\tau )Q(u)\cos [\omega (\tau -u)].$$

We perform the limit *t*→∞ and obtain the expression for the averaged potential energy in the equilibrium state, namely,

33 $${E}_{p}=\mathop{lim}\limits_{t\to \infty }\frac{1}{2}M{\omega }_{0}^{2}\langle {x}^{2}(t)\rangle =\frac{1}{2}M{\omega }_{0}^{2}{\int }_{0}^{\infty }\,d\omega \,{\hat{C}}_{F}(\omega ){I}_{p}(\omega ),$$

where

34 $${I}_{p}(\omega )={\int }_{0}^{{\rm{\infty }}}\,d\tau {\int }_{0}^{{\rm{\infty }}}\,du\,Q(\tau )Q(u)\cos [\omega (\tau -u)]={\hat{Q}}_{L}(i\omega ){\hat{Q}}_{L}(\,-\,i\omega )$$

is the product of a Laplace transform of the response function *Q*(*t*). To obtain the right hand side of this equation, we have exploited relationship between the trigonometric functions and the complex exponential functions (the Euler’s formula), and used the definition (11) for the Laplace transform.

The next step is use the fluctuation-dissipation relation (28) to express the noise correlation spectrum ${\hat{C}}_{F}(\omega )$ by the dissipation spectrum ${\hat{{\rm{\gamma }}}}_{F}(\omega )$. If we insert it to Eq. (33) it becomes

35 $${E}_{p}=\langle {\varepsilon }_{p}\rangle ={\int }_{0}^{\infty }\,d\omega \,{\varepsilon }_{p}(\omega ){{\mathbb{P}}}_{p}(\omega ),$$

where ${\varepsilon }_{p}(\omega )$ defined in Eq. (8) is thermal potential energy per one degree of freedom of thermostat. The function ${{\mathbb{P}}}_{p}$(*ω*) is given by

36 $${{\mathbb{P}}}_{p}(\omega )=M{\omega }_{0}^{2}{\hat{\gamma }}_{F}(\omega ){\hat{Q}}_{L}(i\omega ){\hat{Q}}_{L}(\,-\,i\omega )=\frac{iM{\omega }_{0}^{2}}{\pi \omega }[{\hat{Q}}_{L}(i\omega )-{\hat{Q}}_{L}(\,-\,i\omega )].$$

The right hand side of this equations is obtained in the following way: In the left hand side, we express the Fourier cosine transform (27) by the Laplace transforms (11) for the function ${\hat{\gamma }}_{F}(\omega )=(1/\pi )\,[{\hat{\gamma }}_{L}(i\omega )+{\hat{\gamma }}_{L}(\,-\,i\omega )]$. Next, we use the definition of ${\hat{Q}}_{L}(i\omega )$ and ${\hat{Q}}_{L}(\,-\,i\omega )$ in Eq. (10) and finally, after some algebra, we arrive to the result in (36). In Appendix we show that ${{\mathbb{P}}}_{p}$(*ω*) fulfils all conditions to be a probability measure of some random variable.

### Kinetic energy in an equilibrium state

We proceed in the same way as in the previous subsection: by use of (25) we construct the symmetrized momentum-momentum correlation function (1/2)〈*p*(*t*)*p*(*s*) + *p*(*s*)*p*(*t*)〉, exploit the fluctuation-dissipation relation (28), take *t* = *s* and perform the limit *t*→∞. The final result for the mean kinetic energy in a thermal equilibrium state is

37 $${E}_{k}=\mathop{lim}\limits_{t\to \infty }\frac{1}{2M}\langle {p}^{2}(t)\rangle =\frac{1}{2M}{\int }_{0}^{\infty }\,d\omega \,{\hat{C}}_{F}(\omega ){I}_{k}(\omega ),$$

where

38 $${I}_{k}(\omega )={\int }_{0}^{{\rm{\infty }}}d\tau {\int }_{0}^{{\rm{\infty }}}du\,R(\tau )R(u)\cos [\omega (\tau -u)]={\hat{R}}_{L}(i\omega ){\hat{R}}_{L}(\,-\,i\omega )$$

is the product of a Laplace transform of the response function *R*(*t*). In this equation, we convert the left side to the right side in a similar way as in Eq. (34). Now, we again use the relation (28) to express ${\hat{C}}_{F}(\omega )$ by the dissipation spectrum ${\hat{{\rm{\gamma }}}}_{F}(\omega )$. Then (37) becomes

39 $${E}_{k}=\langle {\varepsilon }_{k}\rangle ={\int }_{0}^{\infty }\,d\omega \,{\varepsilon }_{k}(\omega ){{\mathbb{P}}}_{k}(\omega ),$$

where *ε*_*k*_(*ω*) is thermal kinetic energy per one degree of freedom of the thermostat (see Eq. (8)) and

40 $${{\mathbb{P}}}_{k}(\omega )=\frac{1}{M}{\hat{\gamma }}_{F}(\omega ){\hat{R}}_{L}(i\omega ){\hat{R}}_{L}(\,-\,i\omega )=\frac{1}{\pi }[{\hat{R}}_{L}(i\omega )+{\hat{R}}_{L}(\,-\,i\omega )].$$

We convert the left side to the right side of this equation in a similar way as Eq. (36). In Appendix we prove that this function fulfils all conditions to be classified as a probability distribution of some random variable.

## Appendix

The functions ${{\mathbb{P}}}_{p}$(*ω*) defined by Eq. (36) and ${{\mathbb{P}}}_{k}$(*ω*) defined by Eq. (40) are both probability densities on a positive half-line of real numbers, i.e., they fulfil two conditions:

1. non-negativity, $${{\mathbb{P}}}_{p,k}$$(*ω*) ≥ 0,
2. normalization, $${\int }_{0}^{\infty }\,d\omega \,{{\mathbb{P}}}_{p,k}(\omega )=1.$$

We can prove the non-negativity in the following way. In Eq. (36) and Eq. (40) we use the definitions of ${\hat{Q}}_{L}(\,\pm \,i\omega )$ and ${\hat{R}}_{L}(\,\pm \,i\omega )$ in Eq. (10). For ${\hat{{\rm{\gamma }}}}_{L}(\,\pm \,i\omega )$ in these expressions we apply the relation ${\hat{{\rm{\gamma }}}}_{L}(\,\pm \,i\omega )=A(\omega )\mp iB(\omega )$ with

41 $$A(\omega )={\int }_{0}^{{\rm{\infty }}}\,dt\,\gamma (t)\cos (\omega t),\,\,B(\omega )={\int }_{0}^{{\rm{\infty }}}\,dt\,\gamma (t)\sin (\omega t).$$

Then Eq. (36) and Eq. (40) take the form

42 $$\begin{array}{c}{{\mathbb{P}}}_{p}(\omega )=\frac{2M}{\pi }\frac{{\omega }_{0}^{2}A(\omega )}{{\omega }^{2}{A}^{2}(\omega )+{[M({\omega }_{0}^{2}-{\omega }^{2})+\omega B(\omega )]}^{2}},\\ \,{{\mathbb{P}}}_{k}(\omega )=\frac{2M}{\pi }\frac{{\omega }^{2}A(\omega )}{{\omega }^{2}{A}^{2}(\omega )+{[M({\omega }_{0}^{2}-{\omega }^{2})+\omega B(\omega )]}^{2}}\mathrm{.}\end{array}$$

The denominator in (42) is always positive and it is sufficient to show that the numerator *A*(*ω*) ≥ 0. From Eqs (3), (41) and (27) we deduce that *A*(*ω*) = (*π*/2)*J*(*ω*). From Eq. (5) it follows that *J*(*ω*) ≥ 0 and the same holds true in the thermodynamic limit when *J*(*ω*) becomes a (piecewise) continuous function. Therefore ${{\mathbb{P}}}_{p,k}(\omega )\ge 0$.

The proof of the normalization condition is easier to perform for the distribution ${{\mathbb{P}}}_{k}(\omega )$. From Eq. (9) one can obtain its equivalent form

43 $${{\mathbb{P}}}_{k}(\omega )=\frac{2}{\pi }{\int }_{0}^{{\rm{\infty }}}\,dt\,R(t)\cos (\omega t)={\hat{R}}_{C}(\omega )$$

which is a Fourier cosine transform of the response function *R*(*t*). In turn, its inverse Fourier transform reads

44 $$R(t)={\int }_{0}^{{\rm{\infty }}}\,d\omega \,{\hat{R}}_{C}(\omega )\cos (\omega t).$$

From Eq. (23) it follows that *R*(0) = 1 and for *t* = 0, Eq. (44) reduces to

45 $$R\mathrm{(0)}={\int }_{0}^{\infty }\,d\omega \,{\hat{R}}_{C}(\omega )={\int }_{0}^{\infty }\,d\omega \,{{\mathbb{P}}}_{k}(\omega )=1.$$

So, we proved the normalization of ${{\mathbb{P}}}_{k}(\omega )$. Now, we prove it for ${{\mathbb{P}}}_{p}(\omega )$. From Eq. (36) one can obtain the representation of ${{\mathbb{P}}}_{p}(\omega )$ in the form

46 $${{\mathbb{P}}}_{p}(\omega )=\frac{2M{\omega }_{0}^{2}}{\pi }{\int }_{0}^{+\infty }\,dt\,Q(t)\,\frac{\sin (\omega t)}{\omega }\mathrm{.}$$

By analogy to Eq. (43), we want to find such a function *V*(*t*) that

47 $${\int }_{0}^{+{\rm{\infty }}}\,dt\,V(t)\cos (\omega t)={\int }_{0}^{+{\rm{\infty }}}\,dt\,Q(t)\frac{\sin (\omega t)}{\omega }.$$

The first integral can be rewritten as

48 $${\int }_{0}^{+\infty }\,dt\,V(t)\frac{d}{dt}[\frac{\sin (\omega t)}{\omega }]=-\,{\int }_{0}^{+\infty }\,dt\,\frac{dV(t)}{dt}\,\frac{\sin (\omega t)}{\omega }\mathrm{.}$$

It is true under conditions that *V*(0) is bounded and ${lim}_{t\to \infty }V(t)=0$. Then

49 $$-\frac{dV(t)}{dt}=Q(t)\,\Rightarrow \,V(t)={\int }_{t}^{+\infty }\,Q(\tau )d\tau $$

and it fulfils both conditions. In particular, $V\mathrm{(0)}={\hat{Q}}_{L}\mathrm{(0)}=\mathrm{1/}M{\omega }_{0}^{2}$. From (46) and (47) one gets

50 $${{\mathbb{P}}}_{p}(\omega )=\frac{2M{\omega }_{0}^{2}}{\pi }{\int }_{0}^{+{\rm{\infty }}}\,dt\,V(t)\cos (\omega t)\,\Rightarrow \,V(t)=\frac{1}{M{\omega }_{0}^{2}}{\int }_{0}^{+{\rm{\infty }}}\,d\omega \,{{\mathbb{P}}}_{p}(\omega )\cos (\omega t).$$

For *t* = 0, it reduces to the relation

51 $${\int }_{0}^{+\infty }\,d\omega \,{{\mathbb{P}}}_{p}(\omega )=M{\omega }_{0}^{2}V\mathrm{(0)}=1.$$
